# Tunable electron heating induced giant magnetoresistance in the high mobility GaAs/AlGaAs 2D electron system

**DOI:** 10.1038/srep38516

**Published:** 2016-12-07

**Authors:** Zhuo Wang, R. L. Samaraweera, C. Reichl, W. Wegscheider, R. G. Mani

**Affiliations:** 1 Department of Physics and Astronomy, Georgia State University, Atlanta, GA 30303, USA; 2 Laboratorium für Festkörperphysik, ETH-Zürich, Zürich 8093, Switzerland

## Abstract

Electron-heating induced by a tunable, supplementary dc-current (*I*_*dc*_) helps to vary the observed magnetoresistance in the high mobility GaAs/AlGaAs 2D electron system. The magnetoresistance at *B* = 0.3 T is shown to progressively change from positive to negative with increasing *I*_*dc*_, yielding negative giant-magnetoresistance at the lowest temperature and highest *I*_*dc*_. A two-term Drude model successfully fits the data at all *I*_*dc*_ and *T*. The results indicate that carrier heating modifies a conductivity correction *σ*_1_, which undergoes sign reversal from positive to negative with increasing *I*_*dc*_, and this is responsible for the observed crossover from positive- to negative- magnetoresistance, respectively, at the highest *B*.

Giant magnetoresistance (GMR) denotes a large change in the electrical resistance under the application of a magnetic field and the GMR effect observed in magnetic metallic multilayers (MMM) has now become the canonical GMR effect since it transformed the magnetic hard disk storage and memory industries^1^,^2^,^3^,^4^,^5^,^6^. Physically, the MMM-GMR arises as an applied magnetic field re-aligns the magnetic moments of the successive ferromagnetic layers, which are separated by the nonmagnetic layers^1^,^2^,^5^,^6^. Although not as well known, GMR also occurs in both magnetic and non-magnetic semiconductor systems^3^. Semiconductor GMR is particularly interesting for applications because of the expected ease of integration of associated devices with typical semiconductor electronics^3^. As a consequence, the study of potential new mechanisms for realizing GMR in semiconductors has been a useful line of basic research^7^,^8^,^9^,^10^. Semiconductor GMR in disordered 2D electronic systems has also been a topic of interest from the fundamental physics perspective^11^,^12^,^13^,^14^,^15^,^16^,^17^,^18^,^19^,^20^,^21^,^22^,^23^,^24^,^25^,^26^,^27^,^28^, providing insight into weak localization^11^,^17^, weak anti-localization^11^,^17^, electron-electron interaction-induced magnetoresistance^11^,^14^,^15^,^16^,^18^,^19^,^22^,^23^, metal-insulator transitions induced by a magnetic field^29^, and GMR in the quantum Hall regime^30^,^31^.

Here, we study and model an interesting new mechanism for inducing- and controlling- GMR in a two-dimensional semiconductor system. While previous studies have examined electric field control of magnetoresistance^7^,^9^,^10^, we show that a supplementary dc-current-bias and associated carrier heating in an ac- and dc- current biased high mobility 2DES provides for a current dependent “non-ohmic” decrease in the conductivity with increasing dc current bias in the absence of a magnetic field, and this effect leads to a *dc-current tunable GMR* in the presence of 100’s-of-millitesla-type magnetic fields. Thus, the effect identifies a simple new method for setting the magnitude of the GMR effect as desired, in a semiconductor system.

The ultra high mobility GaAs/AlGaAs system has been the subject of intense magnetotransport studies at high filling factors or low magnetic fields because improvements in material quality in this 2D electron system have led to a steady stream of spectacular new phenomena such as the microwave radiation-induced zero-resistance states and associated magnetoresistance oscillations^32^,^33^,^34^,^35^,^36^,^37^,^38^,^39^,^40^,^41^,^42^,^43^,^44^,^45^,^46^,^47^,^48^,^49^,^50^,^51^,^52^,^53^,^54^,^55^,^56^,^57^,^58^,^59^,^60^,^61^,^62^,^63^,^64^,^65^,^66^,^67^,^68^,^69^,^70^,^71^,^72^, magnetoresistance that depends on the electron-electron interactions^14^,^15^,^16^,^18^,^23^, device size^19^,^21^,^26^, scatterer type^20^,^22^,^24^,^27^, temperature, carrier density^24^, and orientation of magnetic field^25^, giant resonances at the second harmonic of cyclotron resonance^73^,^74^,^75^, etc. The negative magnetoresistance observed in the GaAs/AlGaAs system was initially viewed as a manifestation of disorder-induced electron-electron interaction effect^14^,^15^,^16^. However, features such as the concurrent absence of a Hall-effect correction^26^, dependence upon scattering type^27^, etc., have led to new experimental and theoretical interest. Following upon this interest, we report on an effect which is unexpected within the context of previous theory - a current tunable, carrier-heating-induced negative GMR in the GaAs/AlGaAs 2D system.

## Results

**Figure 1:**
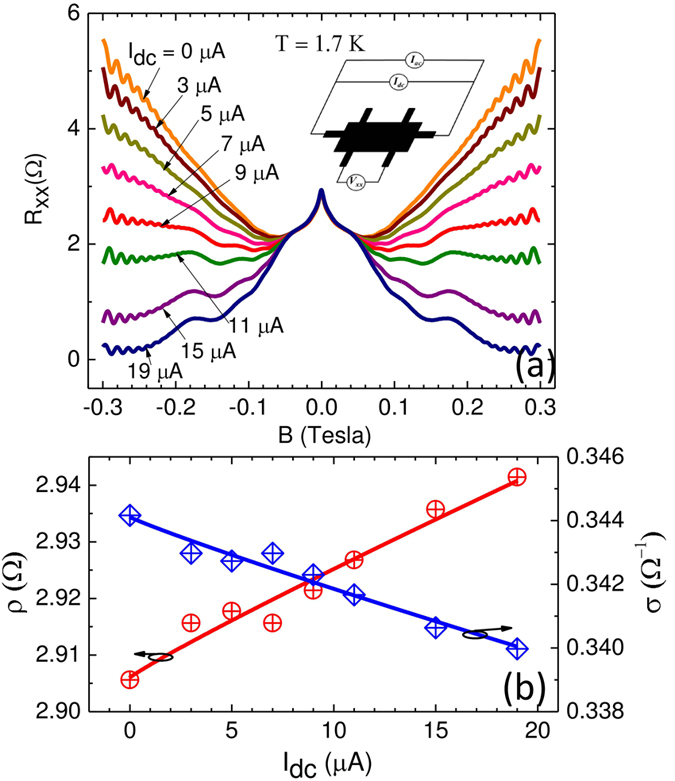
Tunable giant magnetoresistance induced by a dc current bias in a GaAs/AlGaAs heterostructure 2DES. (**a**) Magnetoresistance, *R*_*xx*_, vs. the magnetic field, *B*, at different dc currents. The inset shows a schematic of the measurement. (**b**) The resistivity (red) and conductivity (blue) vs. *I*_*dc*_ in the absence of magnetic field.

Figure 1(a) shows the magnetoresistance at *T* = 1.7 K, as *I*_*dc*_ is varied from 0 to 19 *μ*A. The figure shows that *R*_*xx*_ below *B* = 0.05 T is hardly influenced by the *I*_*dc*_. We found that this part could be simply represented by Δ*R*_*xx*_ = *Aln*(*B*_0_/*B*)^26^. On the other hand, above *B* = 0.05 T, the *R*_*xx*_ vs *B* traces change substantially with the applied *I*_*dc*_. In particular, the observed positive magnetoresistance above *B* = 0.05 T at *I*_*dc*_ = 0 *μA* progressively decreases with increasing *I*_*dc*_ and results in ≥90% negative GMR at *I*_*dc*_ = 19 *μA* and B = 0.3 T. Above *B* = 0.05 T, the magnetoresistance traces also exhibit, to *B* ≈ 0.2 T, magnetophonon oscillations^76^ which increase in amplitude with increasing *I*_*dc*_. This feature, to be examined elsewhere, is one signature of *I*_*dc*_-induced heating. Finally, at the highest *B, B* ≥ 0.2 T, Fig. 1(a) shows Shubnikov de Haas (SdH) oscillations, which appear to be reduced in amplitude with increasing *I*_*dc*_ - yet another signature of *I*_*dc*_ induced electron heating.

In order to convey the variation of the resistivity (*ρ*) and conductivity (*σ*) under the influence of *I*_*dc*_, Fig. 1(b) exhibits the *ρ* (in red) and *σ* (in blue) vs. *I*_*dc*_ at *B* = 0 T. Without heating, one expects *ρ* and *σ* to remain constant under changing *I*_*dc*_, as a consequence of “Ohm’s Law”. The figures show, however, that *σ* is reduced with increasing *I*_*dc*_, roughly by 1.3% for Δ*I*_*dc*_ = 19 *μA*. Thus, the total current-dependent conductivity in the absence of a magnetic field can be written as *σ*(*I*) = *σ*_0_ + *σ*_1_′(*I*_*dc*_), where *σ*(*I*) is the current dependent conductivity. Here, the effect of the ac-current is not included since it is relatively small in magnitude.

**Figure 2:**
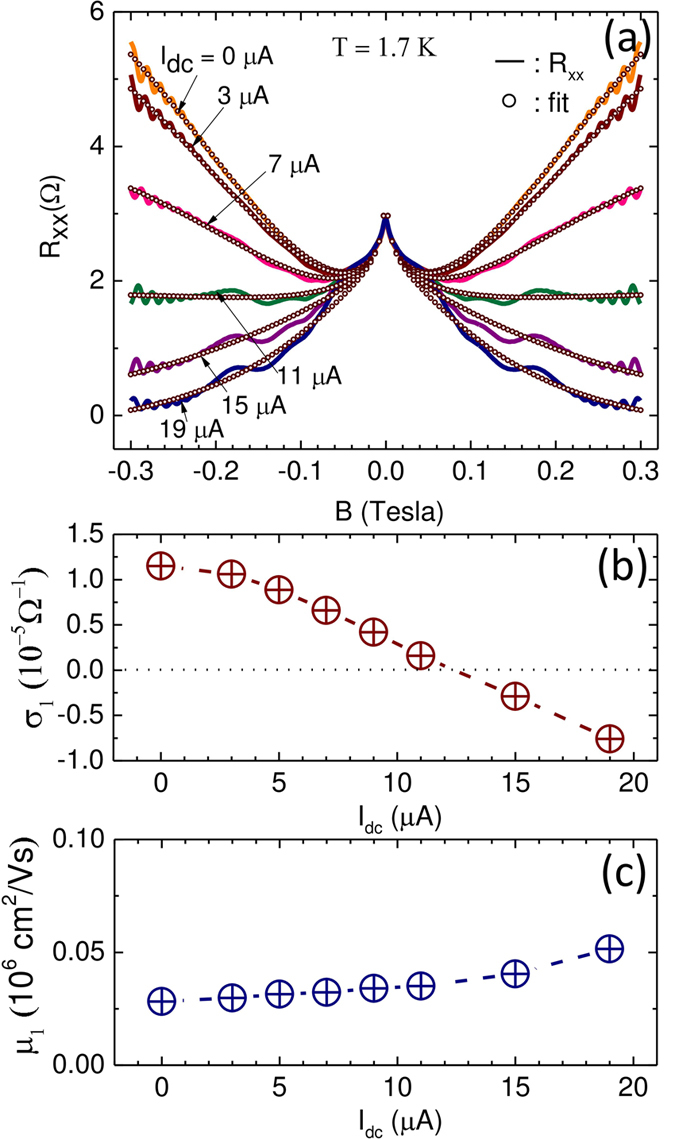
Model fits of the magnetoresistance in a GaAs/AlGaAs 2D electron device. (**a**) Multi-conduction model fits of giant magnetoresistance for various *I*_*dc*_. Solid lines represent data, and symbols represent fit. (**b**) Fit parameter *σ*_1_ vs. *I*_*dc*_. Dashed line is a guide to the eyes. (**c**) Model parametric mobility, *μ*_1_, vs. *I*_*dc*_.

To fit the *B*-dependent GMR results of Fig. 1(a), following previous work^26^, *σ* → *σ*_*xx*_, resulting in 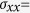

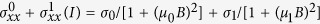
. [Similarly, 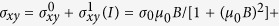

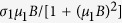
]. Here, *μ*_1_ is a parametric mobility in the Drude model^26^. Since *L*/*W* = 1, we set the diagonal resistance *R*_*xx*_ = *ρ*_*xx*_, the diagonal resistivity, and 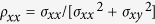 (similarly, the off-diagonal resistivity is 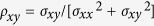). Here, *σ*_0_ = *n*_0_*μ*_0_*e*, where *n*_0_ and *μ*_0_ are the electron density and mobility in the 2D electron system. To account for the *I*_*dc*_ independent magnetoresistance for *B* ≤ 0.05 T, the afore-mentioned additional *ln*(*B*_0_/*B*) term was included. Hence, the magnetoresistance data trace was fit to 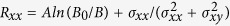. Here, *A* and *B*_0_ were pre-determined by fitting the *B* ≤ 0.05 T data, *n*_0_ is held constant versus *I*_*dc*_ and *T*, while *μ*_0_ is held constant versus *I*_*dc*_ but allowed to vary with *T*. Note that *σ*_1_ and *μ*_1_ are the fundamental parameters that serve to characterize the giant magnetoresistance (GMR) and its change with *I*_*dc*_. Although there are four parameters, *n*_0_, *μ*_0_, *σ*_1_ and *μ*_1_ here, at a given *T*, only *σ*_1_ and *μ*_1_ were allowed to vary with *I*_*dc*_. The fits to the 1.7 K data of Fig. 1(a) are presented in Fig. 2(a), and the fit parameters are summarized in Table (1). Figure 2(a) indicates a good description of the non-oscillatory portion of the data by this empirical fit.

The variation of the fit-obtained parameters *σ*_1_ and *μ*_1_, vs. *I*_*dc*_, are shown in Fig. 2(b) and (c), respectively. Figure 2(b) shows that *σ*_1_ is initially positive, then it gradually decreases, and turns negative above around *I*_*dc*_ = 11 *μ*A while continuing the trend at higher *I*_*dc*_. Thus, *dσ*_1_/*dI*_*dc*_ ≤ 0 recalls the observed *dσ*/*dI*_*dc*_ ≤ 0 in Fig. 1(b). The observed variation in the *σ*_1_ vs. *I*_*dc*_ correlates with the progressive *I*_*dc*_ induced change in *R*_*xx*_ from overall positive- to overall negative GMR to *B* = 0.3 T. Note also that *I*_*dc*_ ≥ 11 *μ*A allows for negative conductivity in *σ*_1_, i.e., *σ*_1_ ≤ 0. Since the parameters in Table 1 suggest that |*σ*_1_| < < |*σ*_0_|, the negative *σ*_1_ is manifested as a resistance correction at *B* = 0 T and a B-dependent (negative) magnetoresistance^26^. Figure 2(c) conveys that the fit parameter *μ*_1_ increases gently with *I*_*dc*_.

**Figure 3:**
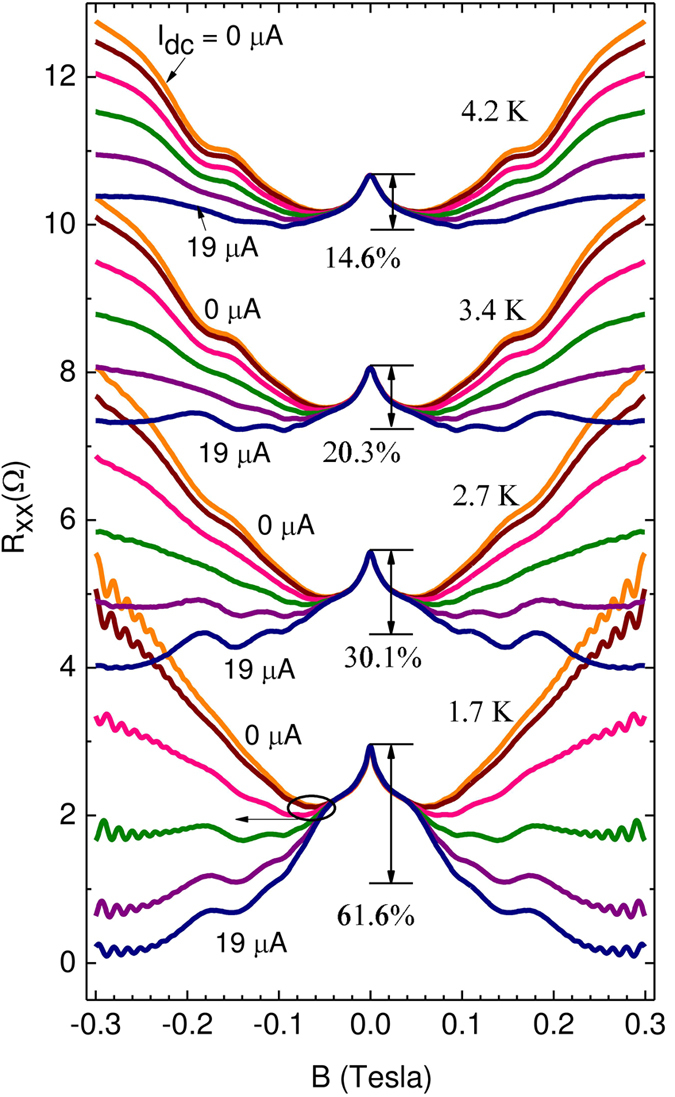
Temperature dependence of the magnetoresistance and its dependence on *I*_*dc*_. This panel shows the *R*_*xx*_ vs. *B*, at various bath temperatures, with the *I*_*dc*_ = 0, 3, 7, 11, 15 and 19 *μA* as the parameter. Here, the data sets at different *T* have been sequentially offset by 2Ω, for the sake of presentation. In addition, the percentage change in the magnetoresistance to B = 0.1 Tesla, at the highest current, is indicated for each temperature.

Similar measurements of *R*_*xx*_ vs. *B* were carried out at higher temperatures. Figure 3 shows *I*_*dc*_ parametrized *R*_*xx*_ vs *B* data-traces at *T* = 1.7 K, 2.7 K, 3.4 K, and 4.2 K, with each data set sequentially offset by 2 Ω. At each *T*, overall positive magnetoresistance is observable at the highest *B* for *I*_*dc*_ = 0 *μA*. However, as *I*_*dc*_ is increased, the positive magnetoresistance is progressively reduced and transformed into negative magnetoresistance at the highest *I*_*dc*_ (the characteristic *T* for this crossover depends on whether or not one includes the weak-localization-like term in the vicinity of *B* = 0 in the consideration). Further, at *T* = 4.2 K, magnetophonon oscillations are apparent at *I*_*dc*_ = 0 *μA* and they are progressive reduced in amplitude with increasing *I*_*dc*_. On the other hand, at *T* = 1.7 K, magnetophonon oscillations are not readily apparent at *I*_*dc*_ = 0 *μA* and become more apparent with increasing *I*_*dc*_. Both these features can be understood as a consequence of the well-known non-monotonic variation of the magnetophonon oscillation amplitude with *T*^76^. That is, while the magnetophonon oscillations vanish in the “low-T” limit, they increase in the amplitude with increasing temperature up to some characteristic temperature, while further temperature increase then leads to a reduction in the oscillation amplitude. Since the 4.2 K traces suggests strong amplitude at *I*_*dc*_ = 0 *μA*, and the increase of *I*_*dc*_ leads to a reduction in the oscillation amplitude, it is apparent that this data set represents the “optimally heated” to the “over-heated” regime, where the magnetophonon oscillation amplitude decreases with a further increase in the temperature, due to the dc-current induced heating. On the other hand, Fig. 3 shows that at 1.7 K, the magnetophonon oscillations are barely perceptible at 1.7 K and they become stronger with increasing *I*_*dc*_. Thus, this data set represents the “under-heated” to “optimally heated” regime, where increasing the temperature with the *I*_*dc*_ increases the oscillation amplitude. Thus, the observed trends in the amplitude of the magnetophonon oscillations also confirm that the *I*_*dc*_ serves to heat the system, as reasoned earlier.

**Figure 4:**
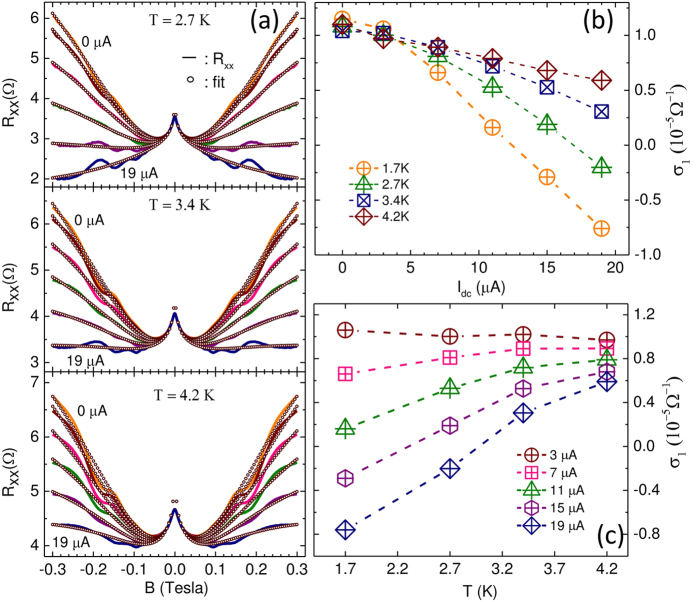
Data fits of the magnetoresistance at different *I*_*dc*_ and temperatures. (**a**) The *I*_*dc*_-dependent magnetoresistance is shown for various *T* with *I*_*dc*_ = 0, 3, 7, 11, 15 and 19 *μA*, along with the empirical data fits. Here, solid lines represent data and symbols represent fits. (**b**) This plot shows *σ*_1_, which is extracted from data fits, vs. *I*_*dc*_, at different temperatures. (**c**) This plot shows *σ*_1_ vs. *T*, at different *I*_*dc*_.

The non-oscillatory part of the *T*-dependent data sets of Fig. 3 were also fit with the multiconduction model; the results are shown in Fig. 4(a) while the fit parameters are summarized in Table 2. Here, symbols represent the fit while the lines represent data. As indicated by Fig. 4, the empirical model succeeds in describing the data at all temperatures and *I*_*dc*_. The extracted fit parameter *σ*_1_ is shown in Fig. 4(b) as a function of *I*_*dc*_. Similar to its behavior at *T* = 1.7 K, the fit extracted *σ*_1_ decreases with increasing *I*_*dc*_ at all *T*. However, the magnitude of the decrease in *σ*_1_ with increasing *I*_*dc*_ is more pronounced at lower *T*. Further, the crossover from positive to negative GMR at *B* = 0.3 *T* is only observed *T* = 1.7 K and *T* = 2.7 K, while at higher *T*, there is only mostly positive GMR. This feature can be correlated with the point that negative magnetoresistance is only observable when *σ*_1_ < 0. As mentioned, the fit extracted *μ*_1_ was only allowed to vary with *I*_*dc*_ but not with *T*. As a consequence, the fit extracted *μ*_1_ vs. *I*_*dc*_ traces at all *T*, not shown, were identical to the Fig. 2(c). Figure 4(c) exhibits the *T*-dependence of *σ*_1_ at various *I*_*dc*_. At small *I*_*dc*_, *σ*_1_ is essentially independent of *T*. On the other hand, at *I*_*dc*_ = 19 *μA, σ*_1_ decreases strongly with decreasing temperature, and the crossover from positive- to negative- *σ*_1_ occurs around *T* = 3 K. Thus, at *I*_*dc*_ = 19 *μA*, negative magnetoresistance due to heating becomes observable below *T* = 3.0 K.

## Discussion

The experimental results shown here in Fig. 1(b) indicate that steady state specimen heating induced by the application of a supplementary *I*_*dc*_ gives rise to a “non-ohmic” current-dependent conductivity with *dσ*/*dI*_*dc*_ ≤ 0. Under the same steady state *I*_*dc*_-induced non-equilibrium conditions, the specimen exhibits a *I*_*dc*_ dependent magnetoresistance to *B* = 0.3 T, which can be successfully described by a two-term Drude model including a second conduction term with the parameters *σ*_1_ and *μ*_1_. Data fitting helps to extract these parameters and shows that *dσ*_1_/*dI*_*dc*_ ≤ 0, similar to *dσ*/*dI*_*dc*_ ≤ 0. While *dσ*/*dI*_*dc*_ ≈ 10^2^Ω^−1^/*A*, the fit parameters show that *dσ*_1_/*dI*_*dc*_ ≈ 1Ω^−1^/*A*. This point suggests that all the conductivity change induced by the dc-current in Fig. 1(b) does not contribute towards the modification of the magnetoresistance described by *σ*_1_ in Fig. 2(b). Thus, it appears that the dc-current modifies *σ* via several modes and not all these modes influence the magnetoresistance. Further, Fig. 4(b) shows that *dσ*_1_/*dI*_*dc*_ varies strongly with *T*. This feature suggests a role for the inelastic scattering length in influencing the GMR, as suggested previously^19^, and its dependence on the *I*_*dc*_ and *T*. The previous fit-study indicated that a negative conductivity term with associated parametric mobility in a multiconduction model is sufficient to realize negative GMR in this 2D electron system^26^. This study shows that the application of *I*_*dc*_ helps to realize such a conductivity correction as a by-product of heating, leading to the observed negative GMR.

Previous theoretical and experimental studies on the effect of steady state heating on the energy distribution of electrons offer insights for our study. For example, experimental photoluminescense studies at a low lattice temperature (e.g., *T*_*L*_ = 10 K) have shown that the photoluminescence lineshape at the fixed lattice temperature broadens and red-shifts with the steady state heating due to terahertz radiation, while lineshape fits utilized to extract an effective (electron) temperature indicate the electron temperature, *T*_*e*_, exceeds the lattice temperature, *T*_*L*_, and the difference *T*_*e*_ − *T*_*L*_ becomes larger with increased terahertz photoexcitation^77^. Remarkably, concurrent studies of the electrical conductivity under the same steady state terahertz drive showed a *monotonic decrease* in the conductivity with increased terahertz photoexcitation which could also be described in terms of an effective (electron) temperature that exceeds *T*_*L*_. Indeed, the *T*_*e*_ determined through the two different experiments showed good agreement. Thus, the energy distribution of electrons under steady state terahertz excitation induced heating could be characterized by an electron temperature that is elevated with respect to the lattice temperature, i.e., *T*_*e*_ > *T*_*L*_^77^. Theoretical studies based on a steady state Boltzmann equation solution of the momentum and energy-balance equations taking into account the electron-LO phonon^78^ and electron-deformation potential acoustic phonon interaction^79^ confirmed that in such steady state drive experiments (i) the electron energy distribution can be characterized by an effective- and elevated- *T*_*e*_, (ii) the electron-phonon interaction dominates both momentum and energy relaxation, (iii) hot electrons relax by emitting or absorbing phonons, which changes phonon number and leads to a phonon number that depends on the electron temperature, and (iv) phonon-drift is negligible^78^,^79^.

Another detailed study of the energy loss mechanism in a low temperature 2DES including ballistic hot electron injection suggested that injecting mono-energetic hot electrons into a 2DES thermalizes the 2D electronic system to a temperature *T*_*e*_ that exceeds *T*_*L*_ when electron-electron scattering rate is large compared to inelastic scattering rate^80^. For hot electron injection energies below the LO phonon energy *E*_*LO*_ = 36meV, all the injected power is transferred via electron-electron scattering to a thermalized 2DES at an elevated *T*_*e*_, and the associated energy is subsequently lost by energy balance to acoustic phonons. In such a situation, *T*_*e*_ is a proportional measure of the injected power, or vice-versa. For hot electron injection energies above the LO phonon energy, injected hot electrons could emit LO phonons before interacting with the 2DES, depending upon the LO phonon emission time, *τ*_*LO*_, and the energy transferred to the 2DES per hot electron could then become the difference between the hot electron energy and the LO phonon energy^80^.

In our experiment, the steady state heating is realized by injecting the supplementary dc current, not by terahertz photoexcitation or by mono-energetic hot electron injection. Yet, the above mentioned results imply that, since we are using the same GaAs/AlGaAs material system in the same low temperature limit, the electron-electron scattering rate likely exceeds the inelastic scattering rate, leading to *I*_*dc*_ heated electron system characterized by a *T*_*e*_ > *T*_*L*_. Further, since the applied supplementary dc currents are modest, *I*_*dc*_ ≤ 19 *μ*A, the effective energies associated with the injected electrons in the supplemental dc current are likely small compared to *E*_*LO*_ = 36 meV. (For *I*_*dc*_ = 19 *μ*A, and an upper bound resistance estimate based the Hall resistance *R*_*xy*_ ≈ 1 *k*Ω at *B* = 0.3 *T*, it turns out that *eIR*_*xy*_ = 19 *meV*, which is less than the *E*_*LO*_ = 36 meV). As a consequence, energy loss is expected to be through acoustic phonons. In this regime, the *T*_*e*_ should increase monotonically with increased *I*_*dc*_. Since electron heating has been shown the reduce the electrical conductivity^77^, and we observe and report a reduction in the conductivity with the increase of the supplementary *I*_*dc*_, while correlating this observation with the characteristic heating-like reduction in the Shubnikov-de Haas oscillation amplitude with increased *I*_*dc*_ (Fig. 1), and the characteristic *I*_*dc*_ induced changes in amplitude of the magnetophonon oscillations (Figs 1 and 3), it is clear that the observed *dσ*/*dI*_*dc*_ < 0 (Fig. 1(b)) is a manifestation of electron heating induced correction to the conductivity, where the *I*_*dc*_ parametrically represents the electron temperature.

When the heating by *I*_*dc*_ produces a “non-ohmic” correction to the conductivity *σ* (Fig. 1(b)), the correction affects the second term in the two-term Drude model modifying, as shown by the modeling presented here, the character of the magnetoresistance. Simply put, the supplementary *I*_*dc*_ heats the electron system, producing a “non-ohmic” decrease in the conductivity. The decrease in the conductivity has the effect of progressively converting the positive magnetoresistance to the negative giant magnetoresistance because the non-ohmic term strongly influences the second term in the Drude model. The magnetoresistance then becomes manifest through the mathematical character of the model.

The practical interest in these results rests upon the possibility of tuning the magnetoresistance in the 2D electron system simply with the application of a supplementary *I*_*dc*_. This suggests the possibility of GMR devices, without a gate-electrode, where the response may be set as desired after manufacture by adjusting dc-current bias with external circuitry. While it is not yet clear which physical channel influenced by the dc-current heating affects the magnetoresistance, these results show that such a channel exists in the 2D electron system. Identification of this channel and the optimization of the physical platform, which are topics for future study, are likely to result in improved control of the giant-magnetoresistance in the 2D electron system.

## Methods

The high-mobility GaAs/AlGaAs heterostructures used in this study were grown by molecular-beam epitaxy (MBE), and patterned into Hall bar devices by photolithography. The 200 *μm* wide Hall bars included voltage probes spaced by 200*μm*, which set the effective Length-to-Width (L/W) ratio *L*/*W* = 1. Electrical measurements were carried out using standard low frequency lock-in techniques. The electron mobility at temperature *T* = 1.7 K was *μ* ≈ 10^7^ *cm*^2^/*Vs* and the density was *n* = 2.4 × 10^11^ *cm*^−2^. The ac- and dc- currents were applied as shown in the inset of Fig. 1. The lock-in sourced ac current source was held constant at 2 *μ*A, as a dc current was varied as desired under computer control. Typically, at a fixed *T*, magnetic field (*B*) sweeps of the lock-in detected diagonal voltage *V*_*xx*_ were collected at a series of constant *I*_*dc*_. The data traces plotted in the Figs 1a, 2a, 3 and 4a report the magnetoresistance 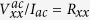. The sample was immersed in pumped liquid helium for these measurements over the range 1.7 K ≤ *T* ≤ 4.2 K.

## Additional Information

**How to cite this article**: Wang, Z. *et al*. Tunable electron heating induced giant magnetoresistance in the high mobility GaAs/AlGaAs 2D electron system. *Sci. Rep.* **6**, 38516; doi: 10.1038/srep38516 (2016).

**Publisher’s note:** Springer Nature remains neutral with regard to jurisdictional claims in published maps and institutional affiliations.

## Figures and Tables

**Table 1:** Fit parameters extracted from empirical data fit at 1.7 K.

*n*_0_(10^11^*cm*^−2^)	*μ*_0_(10^6^*cm*^2^/*Vs*)	*I*_*dc*_(*μ*A)	*σ*_1_(10^−5^Ω^−1^)	*μ*_1_(10^6^*cm*^2^/*Vs*)
2.4	11.56	0	1.15	0.0282
2.4	11.56	3	1.06	0.0298
2.4	11.56	7	0.66	0.0322
2.4	11.56	11	0.16	0.0351
2.4	11.56	15	−0.29	0.0404
2.4	11.56	19	−0.76	0.0515

Here, *n*_0_ and *μ*_0_ are the two-dimensional electron density and mobility, respectively. *σ*_1_ represents the conductivity correction which describes the magnetoresistance and *μ*_1_ represents the associated parametric mobility.

**Table 2:** Summary of the temperature dependence of the fit parameters extracted from the data fits.

*T*(K)	*n*_0_(10^11^*cm*^−2^)	*μ*_0_(10^6^*cm*^2^/*Vs*)	*I*_*dc*_(*μ*A)	*σ*_1_(10^−5^Ω^−1^)	*μ*_1_(10^6^*cm*^2^/*Vs*)
1.7	2.4	11.56	0	1.15	0.0282
			19	−0.76	0.0515
2.7	2.4	9.16	0	1.08	0.0282
			19	−0.20	0.0515
3.4	2.4	7.60	0	1.04	0.0282
			19	0.31	0.0515
4.2	2.4	6.77	0	1.10	0.0282
			19	0.059	0.0515

Here, *n*_0_ and *μ*_0_ are the two-dimensional electron density and mobility, respectively. *σ*_1_ represents the conductivity correction which describes the magnetoresistance and *μ*_1_ represents the associated parametric mobility.
